# Low Toxicological Impact of Commercial Pristine Multi-Walled Carbon Nanotubes on the Yeast *Saccharomyces cerevisiae*

**DOI:** 10.3390/nano11092272

**Published:** 2021-09-01

**Authors:** Sonia Martel Martín, Rocío Barros, Brixhilda Domi, Carlos Rumbo, Matteo Poddighe, Santiago Aparicio, Maria Suarez-Diez, Juan Antonio Tamayo-Ramos

**Affiliations:** 1 International Research Centre in Critical Raw Materials-ICCRAM, University of Burgos, Plaza Misael Banuelos s/n, 09001 Burgos, Spain; smartel@ubu.es (S.M.M.); rbarros@ubu.es (R.B.); bdomi@ubu.es (B.D.); crumbo@ubu.es (C.R.); sapar@ubu.es (S.A.); 2 Laboratory of Materials Science and Nanotechnology (LMNT), Department of Chemistry and Pharmacy, University of Sassari, CR-INSTM, Via Vienna, 2, 07100 Sassari, Italy; m.poddighe16@studenti.uniss.it; 3 Department of Chemistry, University of Burgos, Plaza Misael Banuelos s/n, 09001 Burgos, Spain; 4 Laboratory of Systems and Synthetic Biology, Wageningen University & Research, Stippeneg 4, 6708 WE Wageningen, The Netherlands; maria.suarezdiez@wur.nl

**Keywords:** carbon nanotubes, MWCNTs, *Saccharomyces cerevisiae*, toxicity, oxidative stress, differential expression, transcriptomics

## Abstract

Carbon nanotubes (CNTs) have attracted the attention of academy and industry due to their potential applications, being currently produced and commercialized at a mass scale, but their possible impact on different biological systems remains unclear. In the present work, an assessment to understand the toxicity of commercial pristine multi-walled carbon nanotubes (MWCNTs) on the unicellular fungal model *Saccharomyces cerevisiae* is presented. Firstly, the nanomaterial was physico-chemically characterized, to obtain insights concerning its morphological features and elemental composition. Afterwards, a toxicology assessment was carried out, where it could be observed that cell proliferation was negatively affected only in the presence of 800 mg L^−1^ for 24 h, while oxidative stress was induced at a lower concentration (160 mg L^−1^) after a short exposure period (2 h). Finally, to identify possible toxicity pathways induced by the selected MWCNTs, the transcriptome of *S. cerevisiae* exposed to 160 and 800 mg L^−1^, for two hours, was studied. In contrast to a previous study, reporting massive transcriptional changes when yeast cells were exposed to graphene nanoplatelets in the same exposure conditions, only a small number of genes (130) showed significant transcriptional changes in the presence of MWCNTs, in the higher concentration tested (800 mg L^−1^), and most of them were found to be downregulated, indicating a limited biological response of the yeast cells exposed to the selected pristine commercial CNTs.

## 1. Introduction

Carbon nanotubes (CNTs) have attracted the attention of the scientific community and industry stakeholders due to their wide array of potential applications [1,2], being currently produced and commercialized at a mass scale by large enterprises [3]. Given the impact that new developments based on CNTs are starting to have worldwide, it is essential that the unwanted societal and ecological impacts and risks related to them are determined. Considering the increasing applications, although some studies indicate low bioaccumulation [4], there is a growing likelihood of CNT release into the environment, which could lead to human and ecosystem exposure with potentially harmful effects [5,6]. For this reason, the identification of possible safety issues related to the generation, utilization, and disposal of CNT-based materials is essential, as well as their toxicological assessment, in view of possible biomedical and biotechnological applications [7].

The existence of three CNTs categories has been reported, related to their diameter, consisting of low crystallinity and low specific surface area CNTs (large-diameter multi-walled CNTs (MWCNTs)), low crystallinity and higher specific surface area CNTs (large-diameter single-walled and double-walled CNTs (SWCNTs and DWCNTs)), and high crystallinity and moderate specific surface area CNTs (small-diameter SWCNTs) [8]. MWCNTs offer high tensile strength, elasticity, electrical conductivity, and thermal conductivity, as well as a higher suitability for modification or functionalization compared to other compounds [9], and are the most extensively produced CNT [10]. Despite being reported for the first time 50 years ago [11], novel application fields for this particular type of CNT still keep appearing [12], which also increase the potential appearance of new exposure scenarios [10]. In most cases, carbon nanotube risk assessment studies have been focused on mammalian cell lines and laboratory animals, where mechanisms associated with their potential toxicity have been determined [13]. The biological impact of the nanomaterial has also been studied on microbial systems [14], and a number of studies have explored the toxicity mechanisms based on gene expression analysis in different bacteria [15,16]. MWCNTs seem to have a lower cytotoxicity toward *Escherichia coli* than that induced by single-walled CNTs (SWCNTs), but gene expression data shows that the bacteria overexpressed stress-related gene products in the presence of both CNTs types [16]. In a more recent study, focusing on the transcriptional response of *Pseudomonas aeruginosa* PG201 to different nanomaterials, MWCNT induced a stronger transcriptional response than other nanomaterials of general interest, such as graphene, exfoliated boron nitride, or carbon black [15].

The physiological effects of MWCNTs in fungi have also been evaluated for few species, and these have indicated an ability of the nanomaterial to alter the development of mold structures and their function in organic matter decomposition [17,18]. Studies assessing the response to MWCNTs of the yeast *Saccharomyces cerevisiae*, a model unicellular fungus well-accepted for nanomaterials ecotoxicology assessment [19,20], are also available [21,22], indicating that low MWCNT concentrations have a low impact, while concentrations higher than 100 mg L^−1^ may provoke adverse effects on their viability and proliferation. However, the reported results were based on analyses employing non-commercial grade, low purity (<90%) MWCNTs and oxidized MWCNTs. Additionally, no reports have yet been published analyzing the global cellular response of yeast cells when exposed to the nanomaterial, through omics approaches. Therefore, comprehensive assessments determining the impact of pristine commercial MWCNTs in *S. cerevisiae* cells are still missing. Aiming at filling the highlighted gap, in the present study, pristine commercial MWCNTs were selected and characterized at a physico-chemical and toxicological level, including an analysis of the early global transcriptional response of the yeast, in exposure conditions similar to those employed in previous studies for other carbon derived nanomaterials, for comparative purposes.

## 2. Results and Discussion

### 2.1. Carbon Nanotubes Selection and Characterization

**Figure 1:**
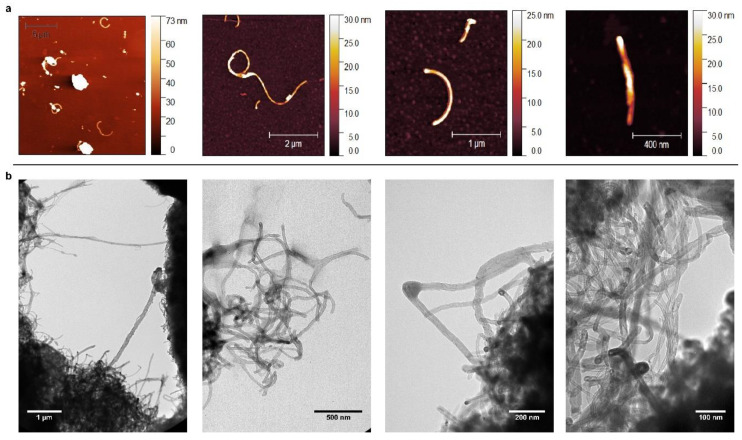
AFM (**a**) and TEM (**b**) images of NTX1 MWCNTs. Samples dispersions with a concentration of 20 mg L^−1^ were deposited by drop casting on a mica surface and carbon-coated copper grids, respectively.

Commercial MWCNTs (NTX1; 97% purity) were obtained from Nanothinx S. A (Patra, Greece). NTX1 CNTs were selected as the production of 100% pure MWCNTs is rare, due to the presence of metal catalysts during the production process [23]. Additionally, 97% purity MWCNTs are of relevance for certain applications due to their metal impurities (iron and aluminum) content, which confer important electromagnetic properties [24]. Prior to their toxicological assessment, the powders were subjected to a physico-chemical characterization. First, microscopy AFM (AC160TS-R3 aluminum reflex coating and tip radius <10 nm; Olympus, Japan) and TEM (JEOL JEM-1011, Japan) analyses were performed, showing that MWCNTs had a diameter in concordance with that described by the provider (15–35 nm), and a variable length in the nm–μm range (Figure 1).

No accurate information was made available by the provider on the elemental composition of NTX1, which can be a very relevant factor influencing the biological response of cells when exposed to MWCNTs [23]. Therefore, the elemental composition of the nanomaterial was analyzed by inductively coupled plasma mass spectrometry (ICP-MS). As shown in Table 1, although the concentration of most of the elements identified was found to be low, significant amounts were detected in some cases, such as Al, Fe, and S. The presence of these elements could be expected, as they are part of the catalysts and substrates employed in the synthesis of CNTs [25,26].

**Figure 2:**
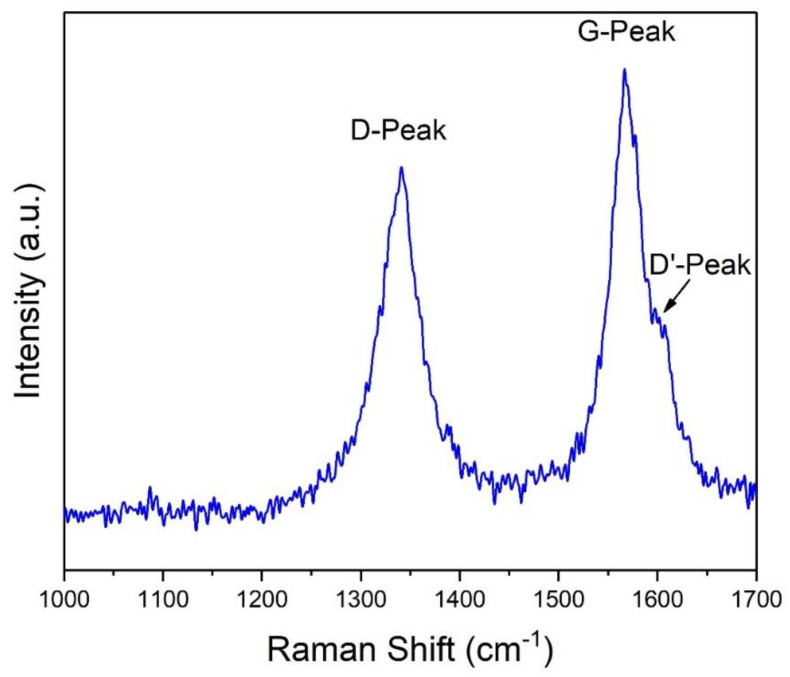
Raman spectra in the range 1000–1700 cm^−1^. Characteristic peak of the crystalline structure of the sample can be observed at 1570 cm^−1^ (G-peak); the peaks at 1339 cm^−1^ and ~1601 cm^−1^ (D-Peak and D’-Peak, respectively) indicate the presence of defects on the surface of the sample.

Nevertheless, considering that CNTs may contain up to 30 wt.% of residual metal impurities after production, and up to 10 wt.% after purification processes [27], it can be assumed that the metal contaminant content of NTX1 CNTs was low [28]. Besides the elemental composition, other physico-chemical aspects, such as defects on their surface, can be determinants of their potential toxicity. To obtain insights into the amount of defects of the selected MWCNTs, a Raman analysis was performed. Figure 2 displays the Raman spectra of NTX1, where the characteristic G and D peaks can be clearly observed. The figure shows the Raman spectra obtained under a laser excitation of 532 nm and a power of 25.0 mW. The peak presents at 1570 cm^−1^ (G-peak) is indicative of the crystalline structure of the sample, while the peak at 1339 cm^−1^ (D-peak) represents defects (or disorder) on the surface of the sample. These defects can be due to a number of factors, such as the presence of sp3 bonds, crystallite boundaries, or the presence of impurities [29,30,31]. The peak at 1570 cm^−1^ has a shoulder at ~1601 cm^−1^, identified as D’-Peak, which is a further indication of the presence of defects on the surface of the sample [32,33,34]. Based on the Lorentzian function, the intensity of the D and G peaks can be calculated, resulting in an ID/IG ratio of ~0.80. This value is intermediate between that of exfoliated graphene (0.02) and that of graphene oxide (1.21) [35,36].

The amount of defects inferred from the observed D-Peak of the selected sample are lower than those observed in other Fe containing MWCNTs previously analyzed at physico-chemical and toxicological levels [31]. Lower defects on CNTs surface have been associated with a higher ability to induce cell viability loss and oxidative stress.

### 2.2. Determination of Viability of S. cerevisiae Cells Exposed to Different Concentration of CNTs

The potential toxicity of the selected CNTs against wild type *S. cerevisiae* BY4741 cells was evaluated by exposing yeast cells to two concentrations of the nanomaterial (160 and 800 mg L^−1^) and two exposure times (2 and 24 h), and performing a colony forming units (CFUs) determination. The selected concentrations are higher than those normally expected in environmentally relevant concentrations, but could occur in exceptional scenarios (e.g., spills, un-controlled waste discharges, etc.) [37,38]. Moreover, the selection was made considering previous reports, which indicated that lower concentrations generate a limited response of the organism [21,22]. Additionally, previous studies assessing the toxicological potential of nanomaterials from different families have been performed employing the same concentrations and exposure conditions, thus allowing a direct comparison of the cellular damage provoked by distinct nanoforms.

**Figure 3:**
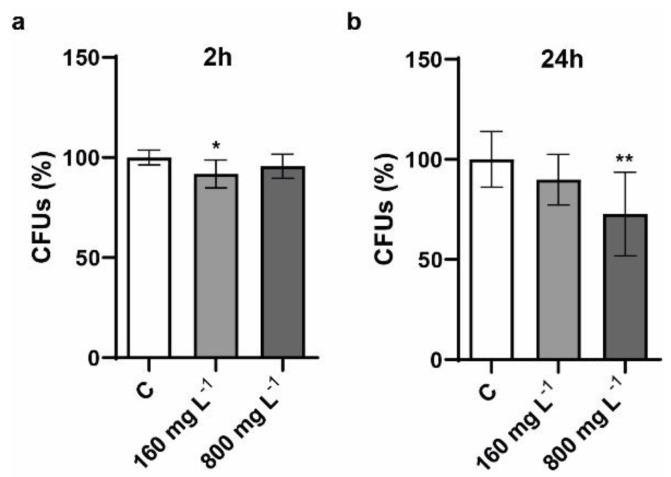
Colony forming unit (CFU) determination of *S. cerevisiae* cells exposed to 160 and 800 mg L^−1^ of CNTs for 2 h (**a**) and 24 h (**b**). The control condition corresponds to non-exposed *S. cerevisiae* cells. The reported values are the averages of nine biological replicates per culture condition. Differences were established using a One-way ANOVA followed by Tukey’s multiple comparisons test, to compare every mean with the control and considered significant at *p* ≤ 0.05. * *p* ≤ 0.05, ** *p* ≤ 0.01.

Colony forming units (CFUs) determination, a standard population quantification method, allowed to measure the potential viability reduction of yeast cells in the presence of NTX1 nanotubes. The exposure conditions were chosen based on those employed in previous research works, allowing a direct comparison of the obtained results with recent studies, were the unicellular fungus was exposed to nanomaterials from different families, such as graphene derivatives, 2D transition metal dichalcogenides (TMDs), or 2D boron nitride [19,22,39,40,41,42]. As displayed in Figure 3, after two hours, CFUs of *S. cerevisiae* exposed to both concentrations were similar to those shown by non-exposed cells. After a longer exposure period (24 h), a significant viability decrease was only observed in the presence of the higher concentration tested (*p* < 0.01). This result is in line with the observations made by Zhu, et al., who observed a notable increase in the mortality rate of *S. cerevisiae* cells exposed to 600 mg L^−1^ of oxidized MWCNTs during 24 h, although smaller concentrations also produced a significant effect on cell proliferation [22]. A previous study, where yeast cells were exposed to non-commercial grade MWCNTs, did not observe an effect on their viability in the presence of concentrations up to 40 mg L^−1^; however, a higher concentrations of 100 mg L^−1^ could provoke cytotoxic effects [21]. Additionally, yeast exposure to SWCNTs over 24 h, at concentrations of 47.1, 94.1, 188.2, and 376.4 mg L^−1^, could reduce cell proliferation and increase mortality [20]. The toxicity provoked in yeast cells after exposure to different concentrations of MWCNTs can be directly compared to that induced by other nanomaterials tested in the same exposure conditions. For instance, graphene oxide monolayer (GO) and graphene oxide nanocolloids (GOC) provoked a viability loss around 50% on *S. cerevisiae* cells after an exposure of 24 h, when the nanomaterials were present at both 160 and 800 mg L^−1^ concentrations [39]. Furthermore, the same concentrations were also tested in recent studies evaluating the potential toxicity of the 2D transition metal dichalcogenides MoS_2_ and WS_2_. In both cases, the viability of *S. cerevisae* was severely reduced when exposed to the selected 2D TMDs, particularly in the presence of MoS_2_ [42,43]. In contrast, graphene and 2D boron nitride nanomaterials showed no ability to reduce yeast cell viability in the same exposure conditions [19,40].

### 2.3. Determination of Oxidative Stress

**Figure 4:**
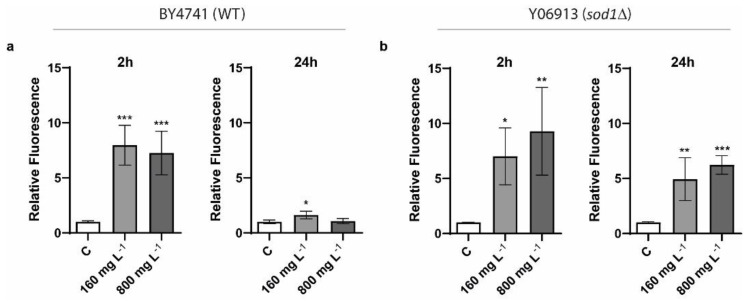
Reactive oxygen species (ROS) levels determination of *S. cerevisiae* cells exposed to 160 and 800 mg L^−1^ of CNTs during 2 h (**a**) and 24 h (**b**) in BY4741 (WT) and Y06913 (*sod1*∆) strains. The control condition (C) corresponds to non-exposed *S. cerevisiae* cells. The reported values are the averages of four biological replicates per culture condition. Differences were established using a One-way ANOVA followed by Tukey’s multiple comparisons test, to compare every mean with the control and considered significant at *p* ≤ 0.05. * *p* ≤ 0.05, ** *p* ≤ 0.01, *** *p* ≤ 0.005.

To evaluate whether the selected commercial CNTs were able to induce oxidative stress in *S. cerevisiae*, strains BY4741 (wild type) and Y06913 (*SOD1* mutant) were exposed to 160 and 800 mg L^−1^ of the nanomaterials, for 2 and 24 h (Figure 4). While the BY4741 strain has an intact antioxidant defense mechanism to cope with oxidative stress, the Y06913 strain contains a null mutation in the *SOD1* gene, encoding a Zn/Cu superoxide dismutase responsible for the detoxification of O_2_^−^, thus playing a role in redox homeostasis. As shown in Figure 4a, intracellular reactive oxygen species (ROS) were significantly higher when BY4741 cells were exposed to both concentrations of the NTX1 powder after 2 h, while ROS levels observed after 24 h in both exposure conditions were almost negligible. The mutant strain showed similar ROS levels to those displayed by the BY4741 after 2 h exposure (Figure 4b). However, in the case of the *sod1*∆ strain, oxidative stress levels were still significantly higher after 24 h exposure, possibly due to its impaired antioxidant defense mechanism, preventing an adequate ROS detoxification similar to that displayed by the wild type strain.

Differently to the observations of Zhu et al., who reported a minor induction of oxidative stress by 600 mg L^−1^ of MWCNTs, in the present study, yeast cells exposed to 160 mg L^−1^ showed a several-fold increase of ROS levels when compared to the negative control. ROS induction was also observed by Zhu et al. in yeast cells when exposed to different concentrations of O-SWCNTs [20]. Additionally, the potential toxic effect of CNTs (MWCNTs and SWCNTs), including the induction of oxidative stress, toward the pathogenic yeast *Candida albicans*, as well as on representative pathogenic bacteria *Staphylococcus aureus* and *Pseudomonas aeruginosa*, showed increased ROS levels in all of the nanomaterial exposure conditions tested (CNT concentration: 100 mg L^−1^; 24 h exposure) [44]. In this regard, it is interesting to highlight that the potential oxidative stress induction and antimicrobial effect of CNTs have been shown to be strongly dependent on several parameters, such as diameter, length, aggregation degree, concentration, surface functionalization, degree of purification, and time and intensity of contact [14]. For instance, previous research suggests that MWCNTs have lower toxicity than SWCNTs towards bacteria, possibly due to the larger diameter of the former [45]. For the same reason, MWCNTs of a smaller diameter exhibit higher cytotoxicity [46]. In any case, taking into account the results obtained in both the CFUs determination and the ROS assay when exposing the BY4741 strain to NTX1 MWCNTs, the toxicity exerted by the selected nanomaterial seems to be small.

### 2.4. Transcriptional Response of S. cerevisiae Cells to Different CN Concentrations

Even if the results described above suggest a low toxicological effect of the selected MWCNTs on *S. cerevisiae*, these are comparable to those reported in a recent study where yeast cells were exposed to graphene nanoplatelets in similar conditions, which unexpectedly induced massive transcriptional changes in the fungal cells upon their exposure to the nanomaterial (160 and 800 mgL^−1^) for a short period of time (2 h) [19]. Therefore, aiming to identify potential sub-lethal toxicity mechanisms, not observed through CFUs and ROS determinations, upon exposure to pristine MWCNTs, a transcriptional analysis was performed to assess the early global response of yeast cells (2 h) to the presence of the nanomaterial (160 and 800 mgL^−1^).

After total RNA isolation and quality control validation via standard procedures (see Materials and Methods), samples were analyzed using the Illumina system. Reads were further mapped to the standard genome BY4741, corresponding to the laboratory strain used in the present study. Information regarding the mapping status can be found in Supplementary Table S1. In all cases, the reads that mapped the *S. cerevisiae* genome ranged between 92.62% and 93.39%, while 85.05% to 88.10% of the reads mapped to exonic regions, giving a good indication concerning the high quality of the RNA generated in this experiment.

**Figure 5:**
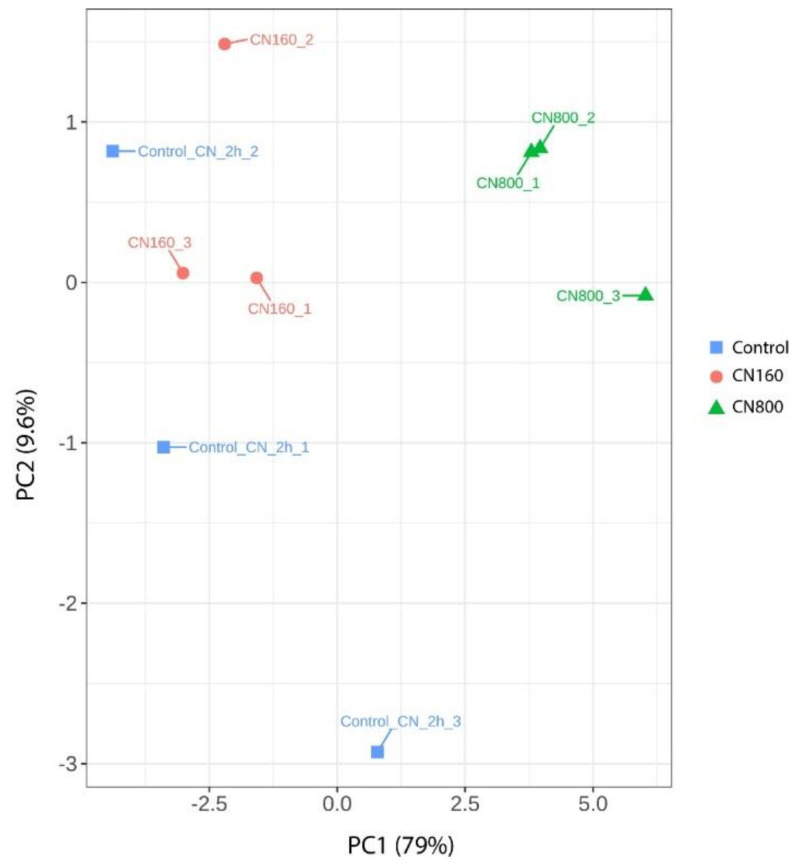
Principal Component Analysis plot of the transcriptomic response of *S. cerevisiae* to two different concentrations (160 and 800 mg L^−1^) of NTX1 MWCNTs and of non-exposed cells (Control). Percentage of the total variance explained by each principal component is indicated on the axis.

Given the high number of dimensions of the obtained data, a principal component analysis (PCA; Figure 5) was performed to observe the variability between the samples considering all expressed genes. Samples corresponding to the control group (untreated cells) and to cells exposed to 160 mg L^−1^ of the nanomaterial are separated from those corresponding to cells exposed to higher concentration (800 mg L^−1^) along the first principal component (PC). The first PC captures 79% of the variability in the full datasets. This indicates that differences between the control (non-exposed cells) and the low exposure is comparable to biological variability among the samples, suggesting a very low effect of the lower concentrations. Differences induced by the exposure conditions are only apparent when the higher concentration is employed.

**Figure 6:**
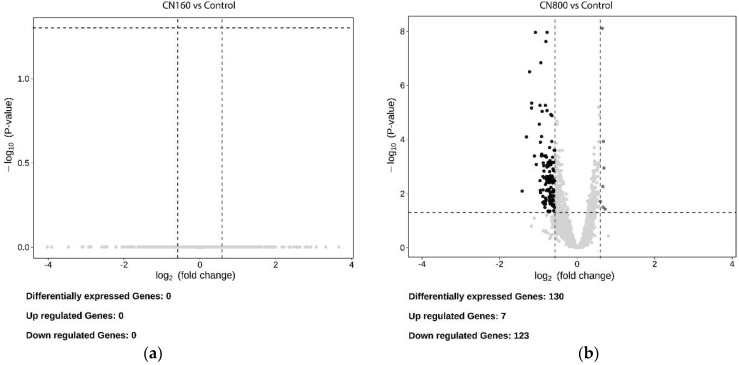
Volcano plots displaying the fold change (log2) of differentially expressed genes of the two conditions, (**a**) CN160 and (**b**) CN800 versus the control. The genes were considered significantly differentially expressed if they had a fold change higher than 1.5 (upregulated) or lower than 1/1.5 (downregulated), and an FDR lower than 0.05.

Regarding the differential expression of genes between CN160 vs. Control and CN800 vs. Control (Supplementary Table S2), volcano plots show clear differences between both exposure conditions in number of genes (Figure 6).

We only consider as differentially expressed with a biological meaning those genes with a difference in expression higher than 1.5-fold (upregulated) or lower than 1/1.5 (downregulated), (corresponding to ±0.585 log_2_ FC), and *p*-value (after correction for multiple testing) lower than 0.05 [47]. It is evident that there are clear differences between both conditions. Regarding the 160 mg L^−1^ exposure condition, no differentially expressed genes could be observed, while a relatively small number of genes (130) showed significant expression changes between the control condition and the 800 mg L^−1^ exposure condition. Supplementary File S1 displays a heat map where the identity and average fold changes of the differentially expressed genes can be observed. The used normalization method (DeSeq2) assumes not all genes to be differentially expressed and introduces sample specific factors per gene to account for, among other things, the possible effects of compositional differences in the transcriptome [48]. Thus, it allows one to explore cases such as the one here presented, were many more genes are downregulated (123) than upregulated (7).

This result is in concordance with that observed in the scattered PCA, which suggests high similarity between the non-exposed cells and 160 mg L^−1^ exposure conditions. Even if the 800 mg L^−1^ exposure condition showed a group of differentially expressed genes in comparison with the control condition, the results obtained in the present work contrast strongly with those observed in the recent study performed by our research group, where yeast cells exposed to 160 and 800 mgL^−1^ of graphene nanoplatelets for 2 h showed strong transcriptional changes (339 and 3591 differentially expressed genes, respectively) with respect to the control condition. Therefore, the transcriptional response of *S. cerevisiae* to carbon nanotubes can be considered significantly weaker than that shown against graphene nanoplatelets [19].

To infer more complex potential toxicity mechanisms, a gene ontology (GO) enrichment analysis was performed of the subsets of upregulated and downregulated genes of the 800 mgL^−1^ exposed condition. GO terms related to biological process, molecular function, and cellular components were tested in the analysis; however, no significantly enriched terms were found among the seven upregulated genes. These are involved in different biological processes, such as metal ion homeostasis: FRE1 (YLR214W), utilization of carbon: HXT4 (YHR092C), nitrogen: ICR7 (YFR055W), and sulfur sources: SAM3 (YPL274W); purine biosynthesis and accumulation: ADE4 and FCY2 (YMR300C and YER056C); and ER-associated protein degradation: MNS1 (YJR131W).

FRE1 produces a cell-surface iron reductase whose overexpression is induced during iron and copper depletion and causes copper sensitivity. Interestingly, the overexpression of iron homeostasis genes in yeast has been reported several times upon exposure to carbon based nanoparticles, such as graphene nanoplatelets and graphene oxide [19,41,49], which have been associated to iron scarcity due to the chelating properties of the nanomaterials. In addition, IRC7, which codes for a cysteine desulphydrase, enabling growth on cysteine as a nitrogen source, is induced in nitrogen and copper limiting conditions [50], while the high-affinity glucose transporter HXT4 has been shown to be overexpressed in low levels of glucose [51]. As discussed in previous studies where transcriptional changes in genes associated with low nutrient availability were observed when *S. cerevisiae* cells were exposed to graphene nanomaterials, their capacity to adsorb biomolecules and ions could lower their availability for biological systems [41].

In regard to possible stress responses that were activated in the presence of NTX1 CNTs, and FCY2, which codes a purine-cytosine permease, is overexpressed upon DNA replication stress [52], and the phosphoribosylpyrophosphate amidotransferase coding gene (YMR300C; ADE4), which is overexpressed in situations of oxidative stress [53], were upregulated in the 800 mg L^−1^ exposure condition.

Amongst the downregulated genes (123), the GO analysis performed did not provide significant results (FDR < 0.05) either. Nevertheless, a large amount of genes that showed significant lower expression levels in yeast cells exposed to 800 mg L^−1^ have functions related to the regulation of transcription and regulation of RNA metabolic processes (YHR206W, YPR070W, YDR423C, YJR017C, YKR077W, YER068W, YML112W, YPR065W, YER120W, YGL194C, YMR043W, YBR083W, YGL237C, YAL013W, YPR018W, YIR018W, YGL035C, YHR041C, YKR095W-A, YDR174W), and ribosome biogenesis (YMR310C, YMR269W, YBL054W, YNR046W), which suggest diminished translational activity. Genes associated to these processes have been observed to be significantly downregulated in previous studies where *S. cerevisiae* was exposed to stress conditions and different toxicants, including carbon derived nanomaterials [19,54,55,56,57]. However, no clear trend was identified in the transcriptional response of yeast cells exposed to the different NTX1 concentrations observed. Only a few differentially expressed genes were identified and only in the presence of the highest concentration, which suggests little response to the material after a short exposure period. [19,54,56,57]. Therefore, considering the toxicological assessment and transcriptomics analysis performed upon exposure of *S. cerevisiae* to commercial pristine MWCNTs, the reported results indicate a limited biological impact of the nanomaterial in the selected ecotoxicity model.

## 3. Materials and Methods

### 3.1. Materials, Reagents and Strains

The chemicals and reagents employed in the present study were purchased from Sigma–Aldrich and Thermo Fisher Scientific. NTX1 MWCNTs were kindly provided by Nanothinxs (Patras, Greece). Working stock suspensions of NTX1 were obtained using ultrapure water, at a final concentration of 1000 mg L^−1^, and were sonicated using a Branson Sonifier Cell Disruptor Model SLPe for 5 min, using an amplitude of 40%. The S. cerevisiae BY4741 strain was purchased from Thermo Fisher. Yeast cells were grown and maintained in standard liquid YPD medium (1% yeast extract, 1% yeast bacto-peptone, 2% glucose). Cell cultures in liquid media were kept on a rotary shaker at 185 rpm at 30 °C.

### 3.2. ICP-MS Analysis

Metal and metalloid content in NTX1 CNTs were determined following the protocol reported by Domi et al., with minor changes [39]. NTX1 samples (0.1 g) were subjected to a digestion process with 7 mL of HNO_3_ Suprapur (Merck) (65% *v*/*v*) and 1 mL of H_2_O_2_ (30% *v*/*v*), while being subjected to the following thermal treatment: a temperature gradient from room temperature up to 80 °C in 4 min, followed by a second temperature gradient, from 80 °C to 120 °C in 4 min, and by a third temperature gradient, from 120 °C to 190 °C in 5 min. Then, temperature was kept constant at 190 °C for 30 min, and finally samples were cooled down for 1 h. The analysis of the digested samples was done with an Agilent 8900 ICP-QQQ instrument (Santa Clara, CA, USA).

### 3.3. Raman Analysis

The Raman measurement was performed by employing a “Senterra” Raman microscope (Bruker) under a laser excitation of 532 nm (25.0 mW power). The spectra was collected with a resolution of ~3–5 cm^−1^, and an integration time of 15 s. The sample (powder) was deposited onto silicon wafer.

### 3.4. Yeast Viability Assays Determination

Yeast cells at OD_600_ = 1 (exponential growth phase) were exposed to 160 and 800 mg L^−1^ of NTX1 for 2 and 24 h, in 1 mL cultures, using 24-well plates. To determine CFUs at both sampling times, 100 μL of cells aliquots, previously diluted 10^4^ times, in the case of 2 h exposure, and 10^5^ times, in the case of 24 h exposure, were spread onto solid YPD medium (6% agar), employing a disposable Digralsky spatula. Subsequently, agar plates were incubated at 30 °C for 48 h. Afterwards, colony forming units were counted for each condition tested. Statistical analyses of the obtained results were carried out using Prism 8.0 (GraphPad Prism, GraphPad Software, Inc., San Diego, CA, USA). One-way analysis of variance (ANOVA) was used for multiple comparisons, followed by Tukey post hoc test. Differences were considered significant at *p* ≤ 0.05.

### 3.5. ROS Determination

To evaluate the yeast intracellular levels of reactive oxygen species after the MWCNT exposure, the CM-H2DCFDA assay was performed, following the protocol described by James et al. [58]. Cells in the exponential phase were pelleted, washed with DPBS (Dulbecco’s phosphate-buffered saline), and then incubated with CM-H2DCFDA (7 μM) in DPBS at 30 °C for 60 min at 185 rpm, protected from light. Subsequently, yeast cells were washed and then resuspended in YPD liquid medium and exposed to the carbon nanotube sample at 160 and 800 mg L^−1^ for 2 and 24 h. Next, yeast cells were washed two times with DPBS, incubated in a solution containing AcLi (lithium acetate) 2 M for 2 min, and successively washed again and incubated in a solution containing SDS (sodium dodecyl sulfate) (0.01%) and chloroform (0.4%) for 2 min. Lastly, yeast cells were pelleted, and the supernatant was transferred to a black opaque 96-micro-well plate. The fluorescence was measured (excitation = 485; emission = 528) using a microplate reader (Synergy-HT, BioTek, Winusky, VT, USA). Statistical analyses of the obtained results were carried out using Prism 8.0 (GraphPad Prism, GraphPad Software, Inc., San Diego, CA, USA). The one-way analysis of variance (ANOVA) was used for multiple comparisons, followed by Tukey post hoc test. Differences were considered significant at *p* ≤ 0.05.

### 3.6. RNA Isolation, Quality Control and Sequencing

RNA isolation was performed using Thermo Fisher Scientific reagents, following the TRIzol Plus RNA Purification Kit user guide (Pub. No. MAN0000561), with the modifications previously described [19]. Briefly, yeast aliquots were pelleted by centrifugation (13,000× *g*) and subsequently resuspended in 1 mL of TRIzol reagent in a 2 mL tube, prefilled with glass beads (MP). Yeast samples were disrupted using a FastPrep-24 Instrument (MP). After disruption, 200 µL of chloroform were added and the mix was homogenated for 10 s. The mix was poured into Phasemaker tubes (2 mL) and centrifuged at 13,000× *g* in a table-top centrifuge. The RNA present in the water phase was purified using the PureLink RNA Mini Kit (Thermo), following the manufacturer’s instructions. RNA integrity was assessed with an Agilent 2100 system, and only high-quality samples (RIN value ≥8) were selected. Total RNA was sent for whole transcriptome sequencing to Novogene Bioinformatics Technology Co. Ltd. (HongKong, China). mRNA sequencing (RNA-Seq) was performed using Ilumina Hiseq4000 and the Casava pipeline version 1.8.2.

### 3.7. RNA-Seq Data Processing and Analysis

Analysis of RNA sequencing data was done following the protocol previously presented [41]. Briefly, read pre-processing for quality filtering was done using FastqPuri [59]. Reads were mapped to the reference genome (accession number GCA_000146045.2), using Star v2.7.2b [60], and retrieved using featureCounts [61]. Total number of reads are summarized in Supplementary Table S1. Data have been submitted to the European Nucleotide Archive and can be found under the accession number PRJEB34524.

Normalization and differential expression calculation was performed using DESeq2 v1.24 [48], with the alpha threshold set to 0.05, and rlog was used for variance stabilizing transformation. Gene ontology enrichment was performed using clusterProfiler v3.12.0 [62] and methods of topGO and DOSE. Annotation files were downloaded from Gene Ontology, release “2019-04-17” and R (version 3.6.0) was used. Additional information concerning each gene was obtained from The *Saccharomyces* Genome Database [63].

## 4. Conclusions

The toxicity assessment of commercial pristine MWCNTs using yeast has unveiled the potential impact of the nanomaterial on the selected model fungus. Overall, the commercial MWCNTs NTX1 were shown to alter the CFU counts of *S. cerevisiae*, only in the presence of high concentrations, while the induced oxidative stress at a short exposure period (2 h) was apparently detoxified at a longer exposure time (24 h). In addition, the analysis of the transcriptional response of yeast cells exposed to different NTX1 concentrations suggest little adaptation or response to the material after a short exposure period, where no clear trend was identified in the transcriptional response of *S. cerevisiae* exposed to the different NTX1 concentrations. Altogether, the reported results indicate a low toxicological impact of the selected pristine commercial MWCNTs on yeast cells in the selected exposure conditions, thus contributing to the understanding of the toxicology and the molecular mechanisms underlying yeast-MWCNTs interactions.

## Figures and Tables

**Table 1:** Inductively coupled plasma mass spectrometry (ICP-MS) analysis of NTX1 MWCNTs. Values below the detection limit of the ICP-MS procedure are also shown. The displayed values are the averages of two independent determinations.

	ppm
Al	8494.29 ± 2523.60
P	7.23 ± 2.05
S	1330.69 ± 45.04
K	83.26 ± 16.93
V	0.04 ± 0.01
Cr	9.35 ± 2.40
Mn	4.30 ± 1.44
Fe	18,986.00 ± 5359.66
Co	0.06 ± 0.02
Ni	0.78 ± 0.09
Cu	1.64 ± 0.33
Rb	<0.001
Sr	<0.001
Zn	0.02 ± 0.02
Nb	0.24 ± 0.07
Mo	4.97 ± 1.38
Ba	<0.001
Ce	0.0016 ± 0.0030
Pr	<0.001
Nd	<0.001
W	<0.001
Pb	<0.001

## Data Availability

The data presented in this study are openly available in the European Nucleotide Archive and can be found under the accession number PRJEB34524.

## Supplementary Materials

The following are available online at https://www.mdpi.com/article/10.3390/nano11092272/s1: Suppementary Table S1, Supplementary Table S2, Supplementary Figure S1.

## Abbreviations

AFM	Atomic Force Microscopy
ICP-MS	Inductively coupled plasma mass spectrometry
TEM RNAseq CNTs MWCNTs DWCNTs	Transition electron microscopy Ribonucleic acid sequencing Carbon nanotubes Multi-walled carbon nanotubes Double-walled carbon nanotubes
SWCNTs	Single-walled carbon nanotubes
SD CM-H2DCFDA	Standard deviation Chloromethyl 2,7-dichlorofluorescin diacetate
ANOVA	One-way analysis of variance
RIN	RNA integrity number
DPBS	Dulbecco’s phosphate-buffered saline
