# Supplementary figures and images for: Low Toxicological Impact of Commercial Pristine Multi-Walled Carbon Nanotubes on the Yeast Saccharomyces cerevisiae
Source: Nanomaterials (Basel). 2021 Sep 1;11(9):2272. doi: 10.3390/nano11092272 (PMC8471963; doi:10.3390/nano11092272)

CN800 vs Control

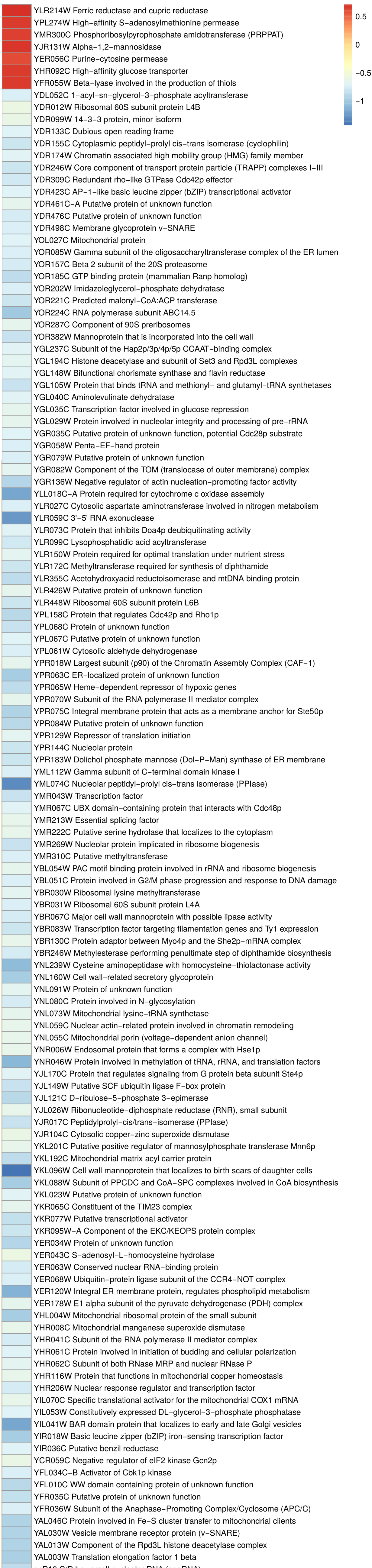

Supplement: Supplementary file 1 [file nanomaterials-11-02272-s001.zip › Supplementary Figure S1.pdf]
